# Serotonin Syndrome Triggered by Overuse of Caffeine and Complicated With Neuroleptic Malignant Syndrome: A Case Report

**DOI:** 10.7759/cureus.22468

**Published:** 2022-02-21

**Authors:** Ryuichi Ohta, Chiaki Sano

**Affiliations:** 1 Communiy Care, Unnan City Hospital, Unnan, JPN; 2 Community Medicine Management, Shimane University Faculty of Medicine, Izumo, JPN

**Keywords:** neuroleptic malignant syndrome, sepsis, general medicine, rural hospital, coffee, caffeine, paroxetine, serotonin syndrome

## Abstract

Serotonin syndrome is a rare complication occurring in patients with psychiatric disorders that are treated with selective serotonin reuptake inhibitors (SSRIs). There are various triggers for serotonin syndrome, including non-SSRI antidepressants. In rare cases, serotonin syndrome may be triggered by nonmedicinal foods, such as coffee. The patient described in this case report was a 65-year-old woman with a past medical history of major depression and a diagnosis of Parkinson’s disease who presented to our medical center with chief complaints of nausea, vomiting, and drowsiness. She had previously been prescribed paroxetine hydrochloride hydrate for depression, and she was prescribed levodopa and carbidopa for Parkinson’s disease. She also drank 20 cups of coffee in a short period of time two days prior to admission due to excessive sleepiness. She was diagnosed with serotonin syndrome based on her clinical symptomology, which included diaphoresis, mydriasis, fine tremor, myoclonus, hypertension, and tachycardia. She was treated with diazepam and cyproheptadine hydrochloride hydrate. Later, she experienced muscle pain with increased creatinine kinase levels after she failed to take levodopa and carbidopa. These findings were suggestive of neuroleptic malignant syndrome. The patient was treated with supportive care. Excessive coffee intake triggers serotonin syndrome by promoting 5-hydroxytryptamine (serotonin) secretion. Patients with psychiatric diseases that necessitate treatment with SSRIs should be educated regarding caffeine consumption. Moreover, patients presenting with agitation and drowsiness should be evaluated for serotonin syndrome within the differential diagnosis. Patients with depression and Parkinson’s syndrome should be evaluated for associated comorbidities, particularly serotonin syndrome and neuroleptic malignant syndrome.

## Introduction

Serotonin syndrome is a rare complication occurring in patients with psychiatric disorders that are treated with selective serotonin reuptake inhibitors (SSRIs). The clinical manifestations of this syndrome vary substantially, from barely perceptible signs to lethal symptomology [1]. More specifically, serotonin receptor overstimulation results in a range of neurological symptoms, including akathisia tremors, altered mental status, clonus, muscular hypertonicity, and hyperthermia [2]. Moreover, the inhibition of serotonin metabolism increases serum serotonin concentrations and triggers overstimulation of central nervous system receptors and peripheral serotonergic receptors [2]. Withdrawal from the identified trigger is the mainstay of treatment for serotonin syndrome [1,2].

There are various triggers for serotonin syndrome. These include SSRI overdosage and the inhibition of serotonin metabolism. SSRI overdosage increases serum serotonin concentrations [3]. Moreover, other substances that moderate serotonin metabolism have been identified in prior research.

Serotonin is produced by the decarboxylation and hydroxylation of L-tryptophan [3,4], and its metabolism is affected by various neurotransmitters, including dopamine, norepinephrine, and monoamine. Because monoamine oxidase (MAO) is essential for metabolism [5], inhibiting MAO leads to an increase in serum serotonin levels, thus resulting in serotonin syndrome [5]. In rare cases, serotonin syndrome is triggered by general nonmedicinal foods (such as coffee) that inhibit MAO. A few prior reports have described cases of serotonin syndrome secondary to caffeine overdose [6].

Serotonin syndrome can coexist with neuroleptic malignant syndrome, which can be challenging to diagnose, especially in severe cases. Serotonin syndrome and neuroleptic malignant syndrome have common clinical symptomology, including hyperthermia and an altered mental status. Herein, we report on a unique case of serotonin syndrome triggered by excessive caffeine intake and complicated with both neuroleptic malignant syndrome and sepsis.

## Case presentation

A 65-year-old woman with a past medical history of major depression presented to our rural hospital with symptoms of nausea, vomiting, and drowsiness that had persisted for one day. Two days prior, the patient began to feel drowsy. She thus self-medicated with 40 cups of coffee over the course of two days, including drinking 20 cups of coffee in a short period of time. The patient had a medical history of major depression, dyslipidemia, and Parkinson’s disease. She took paroxetine hydrochloride hydrate for depression, levodopa/carbidopa for Parkinson's syndrome, and trazodone for insomnia. 

**Figure 1:**
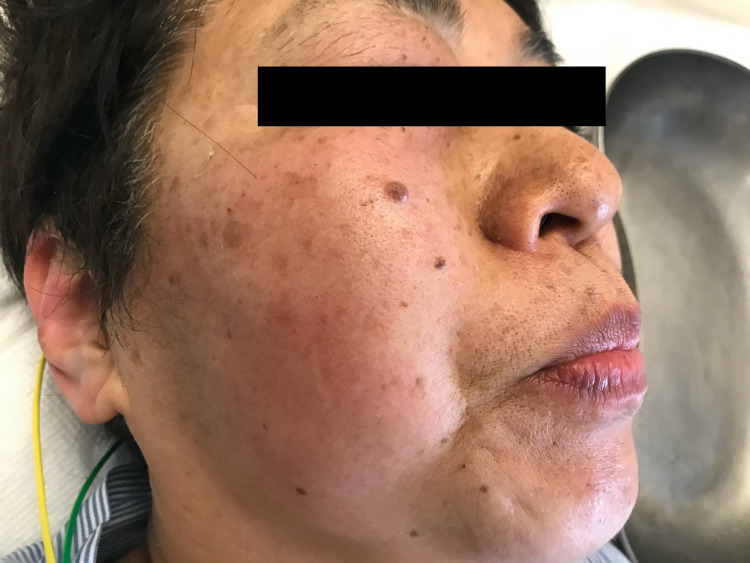
Diaphoretic and hot face.

On initial examination, the patient had a body temperature of 37.2°C, a blood pressure of 165/85 mmHg, a pulse rate of 130 beats/min, a respiratory rate of 30 breaths/min, and a peripheral oxygen saturation (SpO_2_) of 98%. A routine physical examination revealed diaphoresis, mydriasis, fine tremors, myoclonus, hypertension, and tachycardia. Her face was diaphoretic and hot on palpation (Figure 1).

Initial laboratory tests showed an elevated white blood cell count as well as elevated lactate dehydrogenase and creatinine kinase levels (Table 1).

**Table 1:** Laboratory data on admission. PaO_2_: partial pressure of oxygen; PaCO_2_: partial pressure of carbon dioxide.

Marker	Data	Range
White blood cells	19.2	3.5-9.1 × 10^3^/μL
Neutrophils	91.4	44.0%-72.0%
Lymphocytes	3.5	18.0%-59.0%
Monocytes	5.0	0.0%-12.0%
Eosinophils	0.0	0.0%-10.0%
Basophils	0.1	0.0%-3.0%
Red blood cells	3.94	3.76-5.50 × 10^6^/μL
Hemoglobin	12.8	11.3-15.2 g/dL
Hematocrit	38.0	33.4%-44.9%
Mean corpuscular volume	96.4	79.0-100.0 fL
Platelets	31.9	13.0-36.9 × 10^4^/μL
Total protein	7.7	6.5-8.3 g/dL
Albumin	4.9	3.8-5.3 g/dL
Total bilirubin	0.6	0.2-1.2 mg/dL
Aspartate aminotransferase	67	8-38 IU/L
Alanine aminotransferase	39	4-43 IU/L
Alkaline phosphatase	124	106-322 U/L
Lactate dehydrogenase	489	121-245 U/L
Blood urea nitrogen	5.2	8-20 mg/dL
Creatinine	0.57	0.40-1.10 mg/dL
Serum sodium	117	135-150 mEq/L
Serum potassium	3.5	3.5-5.3 mEq/L
Serum chloride	78	98-110 mEq/L
Serum calcium	9.6	3.5-10 mg/dL
C-reactive protein	0.03	<0.30 mg/dL
Creatinine kinase	3579	43-165 U/L
Ferritin	63.7	14.4-303.7 ng/mL
pH	7.410	7.35-7.45
PaO_2_	103.0	60-100 mmHg
PaCO_2_	25.2	35-45 mmHg
Serum bicarbonate	15.6	22-26 mmol/L
Lactate	6.2	0.26-1.39 mmol/L

Based on these clinical findings and according to the previously published Hunter criteria [2], the patient was diagnosed with serotonin syndrome. Paroxetine and trazodone were the suspected triggers. Thus, we discontinued these medications. Her medical conditions were instead treated with diazepam (5 mg) and cyproheptadine (12 mg). Her symptoms were somewhat alleviated following treatment. However, she remained mildly agitated and tachycardic. Thus, she was prescribed continuous intravenous midazolam as well as additional cyproheptadine (up to 18 mg/day) for two days. 

On the second day of admission, she experienced severe muscle pain and her creatinine kinase levels increased to 11,817 mg/dL. We suspected neuroleptic malignant syndrome and malignant hyperthermia within the differential diagnosis. To evaluate this possibility, levodopa and carbidopa were withdrawn for two days. 

The patient was diagnosed with neuroleptic malignant syndrome based on her medical history following the withdrawal of medicine related to dopamine, the observed increase in serum creatinine kinase levels, and her normal CT findings. Her usual medications were then readministered on the second day following admission. She was also provided intravenous intense hydration to prevent acute renal failure. Dantrolene was not prescribed, as her muscle pain improved.

However, her drowsiness and tachycardia persisted as of the fifth day following admission. During the clinical workup for the evaluation of the patient’s etiology, we found that the patient had a body temperature of 37.2°C. Moreover, her urine contained white blood cells and bacteria, which were not detected at admission. The laboratory data showed high C-reactive protein levels (6.60 mg/dL) and a high white blood cell count (17.0 × 103/μL). Based on these findings, the patient was then diagnosed with urosepsis secondary to a urinary tract infection. After being treated with cefmetazole (4 g/day), her mental status recovered. 

Her symptoms gradually improved with antibiotics and supportive care. She was also referred to a psychiatrist and underwent follow-up care for her psychiatric symptoms. On the 14th day following admission, she was discharged directly to her home with minimal symptomology.

## Discussion

This case report demonstrates that serotonin syndrome can be triggered by caffeine overdose. The clinical course of serotonin syndrome differs in healthy patients as compared with those with neurologic disease, including Parkinson’s disease, which can in turn be affected by comorbidities such as neuroleptic malignant syndrome and sepsis.

Patients with psychiatric diseases treated with SSRIs should be educated about the interaction between SSRIs and caffeine. Coffee is a popular beverage that is commonly consumed worldwide. Although the excessive levels of coffee consumption reported in this case are rare, there is no expert consensus regarding the acceptable amount of caffeine intake overall or in relation to serotonin syndrome. Moreover, excessive caffeine consumption is prevalent among psychiatric patients for a variety of reasons [7,8]. In this case report, the patient consumed an extremely large amount of caffeine to reduce drowsiness caused by her prescribed psychiatric medicines (e.g., trazodone) [9]. Since psychiatric patients are more likely to overdose on serotonin from a variety of sources, adequate education with respect to regulating caffeine intake is essential in order to prevent serotonin syndrome and possibly other complications [10].

When managing patients with serotonin syndrome, the coexistence of neuroleptic malignant syndrome should be considered in the differential diagnosis among those presenting with various comorbidities, including Parkinson’s disease. Psychiatric patients often experience multiple cooccurring symptomologies triggered by psychiatric and antidementia medications [11,12]. Because these symptoms are extrapyramidal, reducing the patient’s medication dosage is usually insufficient to address these complications. Thus, dopamine-associated medicines are frequently required [11,12]. Moreover, patients who suffer from acute diseases and cannot take appropriate medicines can develop neuroleptic malignant syndrome [2]. In addition, dopamine stimulates postsynaptic cells, and patients taking dopamine-associated medicines are easily stimulated by serotonin [13]. The usage of both serotonin and dopamine-associated medicines triggers serotonin syndrome [3]. The cascade resulting from these effects can result in neuroleptic malignant syndrome.

Moreover, sepsis should always be included in the differential diagnosis in older patients presenting with drowsiness, altered mental status, signs of high inflammatory activity, and other vague symptomology. In our case, the persistence of low-grade fever, tachycardia, and drowsiness was suggestive of serotonin syndrome. Generally, symptoms of serotonin syndrome present within 24 to 48 hours after the triggering exposure [1,4]. Neuroleptic malignant syndrome is also characterized by short-term symptomology and typically does not present with tachycardia. However, older patients, including those with critical diseases, can present with atypical symptoms [14,15]. Sepsis is diagnosed in serotonin syndrome patients based on their clinical disease course [16]. Laboratory markers of inflammation are helpful in determining the optimal treatment course for older patients [17,18]. Sepsis should be considered in the differential diagnosis among serotonin syndrome patients presenting with persistent tachycardia and elevated inflammatory markers.

## Conclusions

Excessive coffee (caffeine) intake triggers serotonin syndrome by promoting serotonin excretion. Thus, patients with psychiatric diseases treated with serotonin uptake inhibitors should be educated regarding caffeine consumption, especially as this demographic has been shown to frequently consume excessive caffeine. Moreover, those presenting with agitation and drowsiness should be suspected of having serotonin syndrome. Based on our findings and according to the previously published case reports, we conclude that patients with depression and Parkinson’s syndrome should be evaluated for various comorbidities, especially serotonin and neuroleptic malignant syndrome. Our findings guide future research directions and directly inform medical guidelines, health education practices, and effective clinical decision-making.
